# Extracts from Mr. Bernard’s Lecture on Experimental Physiology

**Published:** 1852-03

**Authors:** 


*Extracts from M. Bernard's Lectures on Experimental Physiology.* By J. J. Chisolm, M. D., Charleston.

*Pancreatic Secretion.*—The pancreatic juice, which has long been the object of so *much* speculation and research in the physiological world, has now become known, and proved to be not only a digestive fluid, but the sine qua non, which plays the most conspicuous part in this most important function. Its properties are different from those of any other animal secretion. It is a viscid fluid, nearly limpid, colorless, and always alkaline, in all animals; like albumen coagulating by the application of heat and the mineral or vegetable acids, also deviating light to the left, like that substance; in all of which properties it differs from the saliva, which it was said so closely to resemble. Its perfect coagulability is the test of its good quality. This secretion presents a great variety of faces, according to the circumstances under which it is obtained. The principal precaution necessary, is, that the introduction of the canula for its extraction should be rapid, giving as little pain to the animal as possible. For if the operation is long and tedious, the animal having suffered much, the secretion becomes vitiated, and although alkaline, will not coagulate, and has no action upon animal matters. Therefore, in the fresh drawn fluid, there exists two varieties, according to the difficulties of obtaining it; the one, a good secretion perfectly coagulable by heat, leaving no residual liquid; the other, inert, which is much more limpid, not coagulable and depositing floculi. Between these extremes there is every variety of shade. As the pancreatic juice is very prone to decomposition, a few hours rendering the fluid unfit for experimentation, it should be kept at a low temperature. The good secretion, upon decomposition, deposits needle crystals; the bad none. It is only secreted during digestion, when the gland from congestion appears of a rose color. During fasting, the gland being void of blood, is of a pale color. If the pancreatic juice be collected on the second day after the operation, the quantity will have increased, but so far deteriorated in quality as to have lost all of its properties, on account of inflammation of the gland. For the same reason, in a protracted operation, the gland becomes irritable and the secretion is bad. As a rule, always operate upon animals the least sensible to pain. Among dogs, some varieties, as the common woolly ones, are far preferable to the pointer or setter, which are so sensitive that to obtain a good secretion is impossible. In the horse, whose peritoneum is very sensitive, a good quality cannot be obtained. The same for the rabbit. In the cow, the duct is completely isolated, and being as large as the little finger, the fluid may be collected in large quantity. Birds are very favorable for such experiments, but the ducts being small and multiplied, each from an isolated gland, the collection of the fluid is very tedious. The pancreatic fluid owes its alkalinity to carb. of potash, or soda, the addition of a very dilute acetic acid producing effervescence. The alkaline properties are so very feeble, that they can play no part in the important function of digestion, and if, in fact, it be neutralized by the addition of gastric juice or weak acid, the properties of the secretion remain unchanged. From the facts above related, it is found that the active principle of this secretion resides in the coagulable matter, inasmuch as the absence of this property indicates its want of action upon fat and animal matters. This organic product, which resembles in so many respects albumen, differs from it in this very important particular, viz., that when precipitated by alcohol it is soluble in water and gives to the liquid its characteristic property of forming emulsions, which it will produce in all fats at a sufficiently high temperature. When fats are once mixed with this fluid, they forever after remain in the state of emulsion, whilst when mixed with any of the other animal fluids or secretions, even bile or gastric juice, they soon separate. Upon microscopic examination, the fat is found divided into infinitesimal particles, as in chyle. The mixture gives at first an alkaline reaction, which is, after a little time changed by the fat decomposing, the various acids forming salts, the superabundance remaining free in the mixture. Such is the action of the pancreatic juice externally. But in the process of digestion, these modifications are not produced, as the fat undergoes no chemical changes, but in the state of minute division is absorbed into the lacteal, and may in that state be detected in the thoracic duct, and even in the right chambers of the heart. The fatty matters of the injesta having been melted by the animal temperature of ninety-eight degrees, is immediately upon expulsion from the pylorus acted upon by the pancreatic juice, and absorption commences by the lacteals. This fact is beautifully demonstrated in the rabbit, in which animal the pancreatic duct, instead of emptying, in common with, or in the neighborhood of, the ductus choledochus, into the duodenum, enters the intestine some twelve inches below the pylorus. Fat in a melted state having been introduced into the stomach by means of a catheter, and the animal killed after the lapse of three or four hours, a small quantity of oil was found mingled with the contents of the stomach, not acted on by the gastric juice. The same was found in the duodenum, although freely mingled with the bile; the lacteals retaining the dull white color characteristic of their lymphy contents. But as soon as the pancreatic duct was reached, there an entire change was evident. The contents of the intestine had changed to a milky pultacious mass, the oil had disappeared, the walls of the intestine whitened by the emulsion caused by the fluid appearing through the transparent walls of the villosities, and the lacteals of a milky whiteness presented a striking contrast with those of the superior portion of the canal. This experiment, not only shows in a most satisfactory manner, that the fatty injesta is alone acted on by the pancreatic secretion, and that the chyle is but the addition of minutely divided fat globules to the lymph of the lymphatic system, and that no difference exists between the lymphatics and lacteals, inasmuch as rabbits and similar animals not digesting fat, have no lacteals but all lymphatics, *but* presents to the inquiring mind the fact, that rabbits, which digest no fat, still have a pancreas, which is constantly throwing its secretion into the alimentary canal, to mingle with the injesta. We know that nature would not lumber the economy with a useless organ. But the question naturally arises, what can be the use of this secretion? It is found that when united with the biliary secretion, the great digestive fluid is formed. The bile, as we all know, is composed of several fatty acids, united to bases, forming salts, the most conspicuous of which is the billate of soda. The injesta is, after mastication, carried into the stomach, where it undergoes the action of the gastric juice, which, instead of digesting the matter and rendering them fit for absorption and nutrition, as was formerly believed, only prepares them by a kind of coction to undergo the proper changes at a later period. This gastric juice acting only on the azotized matters, separates the fibrillæ of animal food * by destroying the intercellular tissue, *apparently* dissolving the animal matter into a pultaceous fluid, which, if allowed to settle, deposites a sediment. This, upon microscopic examination, is found composed of the fibrillæ entire not in the least acted on. As soon as this thick fluid is ejected from the stomach, it comes in contact with the bile, which precipitates the albuminous matters. This deposit is called crude chyle, or chyle brute. The same precipitate is produced externally, when to gastric juice which contains dissolved meat, bile is added. Some physiologists suppose that the azotized matters united with the gastric juice, and the biliary salts, undergo a double decomposition, in the presence of each other, viz., the formation of a cholate of albumen, with a lactate of soda; the former is redissolved, and absorbed; the latter is excrementitial. Be this as it may, the acids of bile are readily detected in the precipitate, the other constituent parts passing off in the excrement, billic acid not being detected in fecal matter. The pancreatic juice redissolves the precipitate, and here digestion, properly speaking, commences, the previous intestinal juices having had only a preparatory action upon the injesta. If boiled meats are placed in a mixture of bile and pancreatic juice, they are rapidly dissolved. Raw meats remain unacted upon, the combined fluid dissolving only the fibrillæ, which in raw meat is completely enveloped and protected by a coating of cellular tissue. Boiling converts this intercellular tissue into gelatin, which is soluble in water. The gastric juice, which as before shown, has no action on the fibrillæ, by acting on this cellular substance, prepares the azotized injesta in the same way as coction, so that by a kind of rapid maceration the fibrillæ are so disposed as to be readily brought in contact with the pancreatic juice. For starchy matters, the action is similar; the gastric juice at an elevated temperature, like hot water, being imbibed, swells and ruptures the cell walls of the starchy globules, the fecula

* In Dr. Beamont's experiments, as vegetable matters were the first expulsed from the pylorus, he came to the very false conclusion that they were the soonest digested ; but when he experimented on them out of the stomach, he found that they took much longer time than any other articles of injesta. The reason why these are first expulsed is, that they are not acted on by the gastric juice. Meats alone are acted on, and therefore retained in the receptacle ; all other matters are ejected, before they undergo any marked modification.

escaping, forms a glutinous fluid, which, in the presence of the pancreatic juice, is converted into grape sugar, and is absorbed. What part does the bile alone play? If boiled or raw meat be placed in bile, no change will be produced; meats will keep for an indefinite period. If first macerated in gastric juice, the same will be the condition of things; showing that the action of bile is entirely antiseptic, preventing the decomposition of alimentary matters, and playing only a passive part in digestion. Animal substances decompose freely in the atmosphere, with an evolution of ammoniacal salts. Sugar decomposes into alcohol and carbonic acid, fatty matters into its peculiar acids. Were these same changes to take place in the intestines, the result would be constant diarrhœas, from the irritable properties of these products, as seen when the bile is extracted by a cannula from a dog. The animal dies emaciated and exhausted from diarrhœa. When the pancreatic fluid is mixed with these substances, they all dissolve, showing the digestive properties of the fluid, but a destructive decomposition also goes on at the same time, which is immediately checked by the addition of bile. Thus we find that the three great classes of alimentary matters are each acted on by the pancreatic juice. Fatty matters by emulsion are rendered absorbable. Starchy matters, by their conversion into grape sugar, are rendered soluble and fit for absorption. The same for the albuminous class of injesta. Therefore, as it plays so conspicuous a part in this important function, it richly deserves the appellation of the digestive fluid. Diseases of the pancreas produce the pathognomonic fatty stool, which alone is the sign to detect such organic lesions. Such a pathological state can readily be produced by causing the gland to inflame, slowly, so as either to destroy it completely, or to render it an useless appendage to the economy. Extirpation is impossible, from the intimate relations of the splenic vessels and the extensive and mortal lesions which the peritonium must suffer. Mercury has been injected into the duct, but this acting mechanically as a foreign body produces small abscesses, which soon rupture, discharge their contents, and the foreign irritating body also escaping at the same time, a cure takes place, and after a few days sickness the animal recovers, the organ returning to its natural condition.

M. Bernard, with the ingenuity peculiar to the French experimenter, found that oil injected into the ramifications of the duct in small quantity, ½ oz. for a good sized dog, and the duct, properly speaking, being closed by an injection of suet, which solidifies at the temperature of the body, the desired result is brought about. The oil which fills all of the minute ramifications of the duct, comes in contact with the pancreatic fluid as soon as secreted, an emulsion is formed, which has no means of escape, grace to the barrier of suet, which cuts off all external communication, and as it cannot mingle with the bile which prevents its decomposition, the emulsion becomes converted into the fatty acids, in the interior of the gland, and these, reacting upon the tissues, destroy completely the organ, producing a fibro-amorphous mass, without causing the death of the animal, so gradually are the modifications produced. In the mean time, the appetite of the animal becomes voracious. It eats every thing which comes in its way. But as no digestion can go on, as this fluid is the sine qua non of that important function, the animal soon dies of inanition. The stools have become a greasy mass, and, if examined, will be found isolated, surrounded by an oily stream. Moreover, they are of a pale dirty white color, notwithstanding their free admixture with the bile, showing that this latter secretion alone does not color fecal matter.

After the pancreatic juice has thus acted upon the great mass of injesta, rendering them all in a fit state to be absorbed, what becomes of the pultaceous or heterogeneous compound, the result of these modifications? and by what means is it drawn into the circulation, so as to nourish the body? For fats, we have already seen that they, as an emulsion, are absorbed by the lymphatics of the intestinal canal: and that the formation of chyle is not the great object of digestion, inasmuch as Herbivora, which never eat fat, have no chyle, properly speaking, the contents of the lacteals and lymphatics being identically similar. The same condition of these vessels is seen in Carnivora during fasting. If the emulsion formed from fat and pancreatic juice be added to lymph, a chyle is formed, possessing all of the physical and chemical properties of that contained in the lacteals. Therefore the lymphatics are eclectics, absorbing only fatty matters along with the lymph. True, it is said that they absorb other substances, as seen when charcoal is administered; whilst lampblack or any other impalpable powder infinitely more minutely divided, does not pass into the vessels. The microscope gives a ready solution of this fact. The particles of charcoal are found covered with angles and asperities, which readily cut their way through the delicate coats of the vessels, and are hurried off in the circulation, whilst the lampblack is found composed of globules. The other alimentary products, sugar and albumen, are both rendered soluble, and are absorbed by the portal venous system, and can be readily detected in the viscera veins. They are then passed through the liver, which is the great assimilating organ rendering them fit for nutrition, and converting dead matter into living man.

A most singular fact connected with alimentary absorption is, that the coats of the intestine become satiated, when but one substance is used as injesta, and refuses to absorb it. This is exemplified by feeding a dog on albumen alone. At first it is all absorbed and nourishes the animal. This quantity absorbed rapidly diminishes, and finally albumen making its appearance in the stools, it will be found to pass by stool, the quantity in the excrement being the same by weight or measure as the quantity of injesta. Not a particle having been absorbed, the intestine revolts; the dog dies of starvation, although the most nutritious of aliments is given in abundance. If potash is given to animals, at first it will be absorbed and pass off by the urine, but after a certain time, it will disappear from this excretion and will be found in the fæces. The same is the case of all substances. If an exometer be formed of a piece of membrane, covering a glass tube, in which a dense solution is placed, and the whole be injected into a vase containing a mixture of potash, the salt will pass through for eighteen or twenty hours, when all endosmotic action of the salt will stop, the membrane having lost temporarily all power of absorption over this substance. But if a new salt be added, then the endosmotic action of the new substance immediately commences. This property is, therefore, not vital but inherent in the membrane.—*Charleston Medical Journal and Review.*

